# Characterising the Intestinal Bacterial and Fungal Microbiome Associated With Different Cytokine Profiles in Two *Bifidobacterium strains* Pre-Treated Rats With D-Galactosamine-Induced Liver Injury

**DOI:** 10.3389/fimmu.2022.791152

**Published:** 2022-03-24

**Authors:** Hua Zha, Qian Li, Kevin Chang, Jiafeng Xia, Shengjie Li, Ruiqi Tang, Lanjuan Li

**Affiliations:** ^1^ State Key Laboratory for Diagnosis and Treatment of Infectious Disease, Collaborative Innovation Center for Diagnosis and Treatment of Infectious Diseases, National Clinical Research Center for Infectious Diseases, The First Affiliated Hospital, Zhejiang University School of Medicine, Hangzhou, China; ^2^ Department of Statistics, The University of Auckland, Auckland, New Zealand

**Keywords:** intestinal microbiome, bacteria, fungi, probiotics, liver injury

## Abstract

Multiple probiotics have protective effects against different types of liver injury. Different intestinal microbes could be beneficial to the protective effects of the probiotics on the treated cohorts in different aspects. The current study was designed to determine the intestinal bacterial and fungal microbiome associated with different cytokine profiles in the *Bifidobacterium pseudocatenulatum* LI09 and *Bifidobacterium catenulatum* LI10 pretreated rats with D-galactosamine-induced liver injury. In this study, partition around medoids clustering analysis determined two distinct cytokine profiles (i.e., CP1 and CP2) comprising the same 11 cytokines but with different levels among the LI09, LI10, positive control (PC), and negative control (NC) cohorts. All rats in PC and NC cohorts were determined with CP1 and CP2, respectively, while the rats with CP1 in LI09 and LI10 cohorts had more severe liver injury than those with CP2, suggesting that CP2 represented better immune status and was the “better cytokine profile” in this study. PERMANOVA analyses showed that the compositions of both bacterial and fungal microbiome were different in the LI10 cohorts with different cytokine profiles, while the same compositions were similar between LI09 cohorts with different cytokine profiles. The phylotype abundances of both bacteria and fungi were different in the rats with different cytokine profiles in LI09 or LI10 cohorts according to similarity percentage (SIMPER) analyses results. At the composition level, multiple microbes were associated with different cytokine profiles in LI09 or LI10 cohorts, among which *Flavonifractor* and *Penicillium* were the bacterium and fungus most associated with LI09 cohort with CP2, while *Parabacteroides* and *Aspergillus* were the bacterium and fungus most associated with LI10 cohort with CP2. These microbes were determined to influence the cytokine profiles of the corresponding cohorts. At the structure level, *Corynebacterium* and *Cephalotrichiella* were determined as the two most powerful gatekeepers in the microbiome networks of LI09 cohort CP2, while *Pseudoflavonifractor* was the most powerful gatekeeper in LI10 cohort with CP2. These identified intestinal microbes were likely to be beneficial to the effect of probiotic *Bifidobacterium* on the immunity improvement of the treated cohorts, and they could be potential microbial biomarkers assisting with the evaluation of immune status of probiotics-treated cohorts.

## Introduction

Liver injury has caused great illness in human beings (1). It could be induced by multiple factors, e.g., drug, virus, alcohol, food additives, and dietary supplements (2–5). A variety of products and materials have been determined to have protective effects against different types of liver injury (6–8).

The protective effects of probiotics against liver injury have been widely reported. For example, *Lactobacillus plantarum* C88 was capable of preventing ethanol-induced mice liver injury (9). *Bacillus spores* could protect rats from acetaminophen-induced acute liver injury (10). *L. plantarum* C88 was found to protect mice from aflatoxin B1-induced liver injury (11).

Different intestinal microbes were likely to work in concert with probiotics to promote health. *Bifidobacterium pseudocatenulatum* LI09 and *Bifidobacterium catenulatum* LI10 were found to alleviate the severity of D-galactosamine (D-GalN)-induced rat liver injury (12). However, the intestinal microbes that can enhance the protective effects of the two *Bifidobacterium* on the improvement of cytokine profiles have not been determined.

The current study was designed to (1) characterize the intestinal bacterial and fungal microbiome associated with different cytokine profiles of LI09 and LI10 pretreated rats with D-GalN-induced liver injury and (2) investigate the microbes with the biomarker potentials to assist with the evaluation of better immune status in the probiotics-treated cohorts.

## Materials and Methods

The animal experiments were performed as previously described (12), with a few modifications. Briefly, *B. pseudocatenulatum* LI09 and *B. catenulatum* LI10 were streaked on the trypticase–phytone–yeast agar and revived anaerobically at 37°C. The two bacterial strains were then prepared in physiological saline (PBS) at a final concentration of 3 × 10^9^ CFU/ml. Sprague–Dawley male pathogen-free rats weighting 250–350 g were fed with a standard laboratory rat chow and raised under the 12:12 light/dark cycle at 22°C for 7 days to adapt to the environment.

The 122 rats were randomly allocated into four cohorts, i.e., LI09 cohort (n = 40), LI10 cohort (n = 40), positive control (PC) cohort (n = 22), and negative control (NC) cohort (n = 20). The rats in LI09 and LI10 cohorts were orally administrated with a 1-ml aliquot of LI09 or LI10 (3 × 10^9^ CFU) for a week, while the rats in PC and NC cohorts were orally administrated with 1-ml aliquot of PBS for the same period. Afterwards, an intraperitoneal injection of D-GalN was given to each of the rats in LI09, LI10, and PC cohorts at a dose of 700 mg/kg body weight. Twenty-four hours after the induction of liver injury, all the living rats were anesthetized by an intraperitoneal injection of 10 mg/kg xylazine and 80 mg/kg ketamine, before being subjected to laparotomy for collection of the blood, liver, and cecal content. The study was approved by Animal Care and Use Committee of the First Affiliated Hospital, Zhejiang University School of Medicine.

### Measurement of Liver Function Variables

Serum was extracted from blood samples by centrifugation and stored at −80°C. Concentrations of liver function variables in serum, i.e., gamma glutamyl transferase (GGT), total bilirubin (TB), total bile acid (TBA), albumin (ALB), aspartate aminotransferase (AST), alanine aminotransferase (ALT), and alkaline phosphatase (ALP), were measured by an automatic biochemical analyzer (Roche Diagnostics, Ottweiler, Germany) according to the manufacturer’s instructions.

### Measurement of Serum Cytokines

The concentrations of 23 cytokines in the serum samples were measured using a Bio-Plex Pro™ Rat Cytokine 23-Plex Assay kit (Bio-Rad Ltd., Hercules, CA, USA) as per the protocol of the manufacturer. These cytokines included macrophage inflammatory protein (MIP)-1α, MIP-3α, macrophage colony-stimulating factor (M-CSF), granulocyte colony-stimulating factor (G-CSF), granulocyte–macrophage colony-stimulating factor (GM-CSF), interferon gamma (IFN-γ), tumor necrosis factor alpha (TNF-α), interleukin (IL)-1α, IL-1β, IL-2, IL-4, IL-5, IL-6, IL-7, IL-10, IL-12p70, IL-13, IL-17A, IL-18, monocyte chemoattractant protein-1 (MCP-1), growth-related oncogene (GRO)/keratinocyte chemoattractant (KC), regulated upon activation, normal T cell expressed, and secreted (RANTES), and vascular endothelial growth factor (VEGF).

### Evaluation of Liver Injury Severity

The liver tissue from the left liver lobe of each rat was dissected and fixed in 10% formalin solution, before being dehydrated and processed in paraffin using standard histological methods. The liver samples were stained and mounted on microscope slides. The liver injury severity was evaluated by a professional pathologist based on the Ishak scoring system (13).

### Molecular Experiments

DNA was extracted from the cecal samples by using a DNeasy PowerSoil kit (MoBio Laboratories Inc., Carlsbad, CA, USA). The extracted DNA was respectively amplified with bacterial primers (i.e., 341F/785R) and fungal primers (i.e., ITS3F/ITS4R) (14, 15). The PCR products were purified by using a DNA Clean and Concentrator Kit (Zymo Research, Irvine, CA, USA), and their concentrations were measured by using a Qubit™ dsDNA HS Assay Kit (Thermo Fisher Scientific Inc., Waltham, MA, USA). The purified PCR products were submitted for sequencing on Illumina NovaSeq 6000 platform (Illumina Inc. USA).

### Processing of Sequencing Data

The sequencing data were processed with standard bioinformatic procedures, e.g., merge, quality filter, singleton removal, and chimera check. The sequences were clustered into groups of amplicon sequence variants (ASVs). Bacterial phylotypes were classified using QIIME2 against the Silva 138 database, while fungal phylotypes were classified against the UNITE fungal database. One rat in the LI10 cohort was not recruited in the current study, as it did not provide enough sequencing reads. All the other rats were recruited for the subsequent analyses.

### Cytokine Profile Analyses

One-way ANOVA tests and Mann–Whitney tests were used to determine the differences in 23 cytokines in LI09, LI10, PC, and NC cohorts. The cytokines with significant differences among the four cohorts were selected and transformed in log_2_(X+1) for the cytokine profile analyses.

Partition around medoids (PAM) clustering analysis was performed to cluster the cytokine profiles of all the four cohorts, after an average silhouette analysis being conducted to determine the optimal number of clusters. The four cohorts were determined with two distinct cytokine profiles, i.e., CP1 and CP2. The rats in LI09 and LI10 cohorts with two different cytokine profiles were defined as CP1_LI09, CP2_LI09, CP1_LI10, and CP2_LI10 cohorts.

### Comparisons of Liver Function Variables and Ishak Scores

T-tests and Mann–Whitney tests were used to compare the liver function variables in CP1_LI09 and CP2_LI09 cohorts. The same tests were performed to compare the liver function variables in CP1_LI10 and CP2_LI10 cohorts.

Mann–Whitney tests were carried out to compare the Ishak scores of CP1_LI09 and CP2_LI09 cohorts and those of CP1_LI10 and CP2_LI10 cohorts.

### Bacterial and Fungal Microbiome Composition Analyses

The alpha diversity indices (i.e., observed species and Shannon and Pielou indices) of the bacterial and fungal microbiome in the LI09 cohorts with different cytokine profiles, and their control cohorts (i.e., PC and NC cohorts), were all calculated. One-way ANOVA was used to compare the alpha diversity indices of the four cohorts, and t-tests were performed for the comparisons of LI09 and control cohorts. The same strategy was carried out for the same microbiome composition comparisons of LI10 cohorts with different cytokine profiles and their control cohorts.

Permutational analysis of variance (PERMANOVA) was conducted in R 4.1.0 to compare the CP1_LI09 and CP2_LI09 cohorts for their bacterial and fungal microbiome compositions and compare them with their control cohorts (i.e., PC and NC cohorts). The same strategy was performed for the same microbiome composition comparisons of CP1_LI10 and CP2_LI10 cohorts and their control cohorts.

Similarity percentage (SIMPER) analysis was performed to determine the bacterial and fungal microbiome similarities within the LI09 and LI10 cohorts with different cytokine profiles. The same analysis was used to determine the bacterial and fungal microbiome dissimilarities between CP1_LI09 and CP2_LI09 cohorts. The same strategy was used for the comparisons of the bacterial and fungal microbiome dissimilarities between CP1_LI10 and CP2_LI10 cohorts.

Linear discriminant analysis (LDA) effect size (LEfSe) was carried out to compare the bacterial and fungi microbiome of CP1_LI09 and CP2_LI09 cohorts, respectively, to determine the bacteria and fungi associated with each of the two cohorts. The same analysis was performed to determine the bacteria and fungi associated with CP1_LI10 and CP2_LI10 cohorts.

### Effects of Bacteria and Fungi on the Cytokine Profiles

Spearman test was used to determine the individual correlations between the cytokines in the cytokine profiles and the microbes associated with CP1_LI09, CP2_LI09, CP1_LI10, and CP2_LI10 cohorts.

Distance-based redundancy analysis (db-RDA) was performed to determine the effect of bacteria and fungi associated with CP1_LI09, CP2_LI09, CP1_LI10, and CP2_LI10 cohorts on the corresponding cytokine profiles.

### Correlations Between Bacteria and Fungi

Spearman test was carried out to investigate the correlations between the bacteria and fungi associated with CP1_LI09 cohort. The same strategy was performed for the bacteria and fungi correlations in CP2_LI09, CP1_LI10, and CP2_LI10 cohorts.

### Microbiological Network Analyses

The correlations between the bacteria and fungi in the intestinal microbiome networks of LI09 and LI10 cohorts with different cytokine profiles were determined by co-occurrence network inference (CoNet) analysis. Five metrics, i.e., Bray–Curtis, Spearman, Pearson, mutual information, and Kullback–Leibler dissimilarities, were used to calculate the ensemble inference in CP1_LI09, CP2_LI09, CP1_LI10, and CP2_LI10 cohorts. The top 10 microbes (i.e., bacteria and fungi) with the largest numbers of correlations in the microbiome networks of the four cohorts were determined.

Network fragmentation calculations and generation of a null distribution were carried out in R as previously described (16) to explore the gatekeeper(s) in the microbiome networks of LI09 and LI10 cohorts with different cytokine profiles. Statistical significance was defined as the number of times a fragmentation score over that resulting from the removal of the bacteria or fungi observed within the null distribution.

## Results

### Cytokine Profile Analysis

Eleven serum cytokines were determined with significant differences among the LI09, LI10, PC, and NC cohorts, i.e., IL-1α, IL-2, IL-4, IL-5, IL-6, IL-12p70, IL-17A, M-CSF, MCP-1, MIP-3α, and RANTES (all *p* < 0.03), and they were used for the subsequent clustering analysis of cytokine profiles. Higher levels of IL-1α and M-CSF were determined in both LI09 and LI10 cohorts than in PC cohort, and IL-2 and IL-17A were greater in the LI10 cohort than in the PC cohort (**Supplementary Figure S1**), suggesting IL-1α, M-CSF, IL-2, and IL-17A were enhanced by LI09 and/or LI10. In contrast, MCP-1, IL-5, and MIP-3α were determined with lower levels in both LI09 and LI10 cohorts than in the PC cohort (**Supplementary Figure S1**), suggesting that they were suppressed by LI09 and LI10.

**Figure 1:**
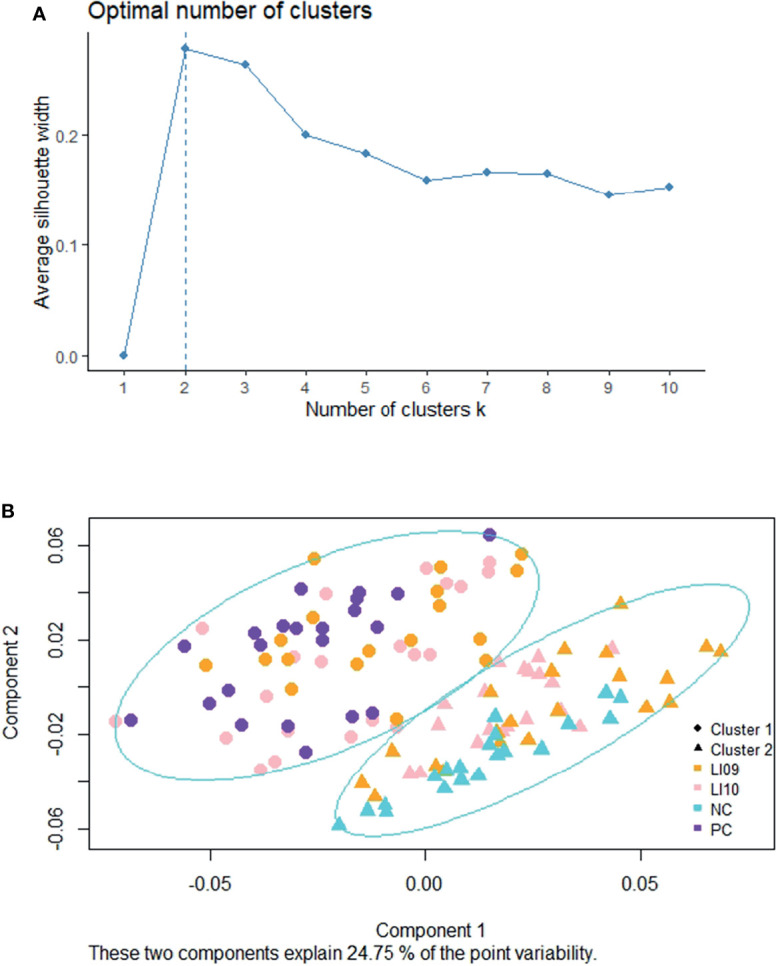
Clustering of cytokine profiles in LI09 and LI10 cohorts. **(A)** Silhouette analysis determined the scores for the optimal clusters. **(B)** Partition around medoids clustering analysis determined the two cytokine profiles (i.e., CP1 and CP2) in all the recruited cohorts.

Two was determined as the most optimal number for clustering the cytokine profiles (**Figure 1A**), and two distinct cytokine profiles (i.e., CP1 and CP2) were determined in the LI09, LI10, PC, and NC cohorts (**Figure 1B**). All rats in the PC cohort were determined with CP1, and all rats in the NC cohort had CP2, suggesting that CP2 represented better immune status and was the “better cytokine profile.” Twenty-one rats in the LI09 cohort and 18 rats in the LI10 cohort were determined with CP1, while 19 rats in the LI09 cohort and 21 rats in the LI10 cohort had CP2 (**Figure 1B**).

### Liver Function and Liver Injury Severity Analyses

**Figure 2:**
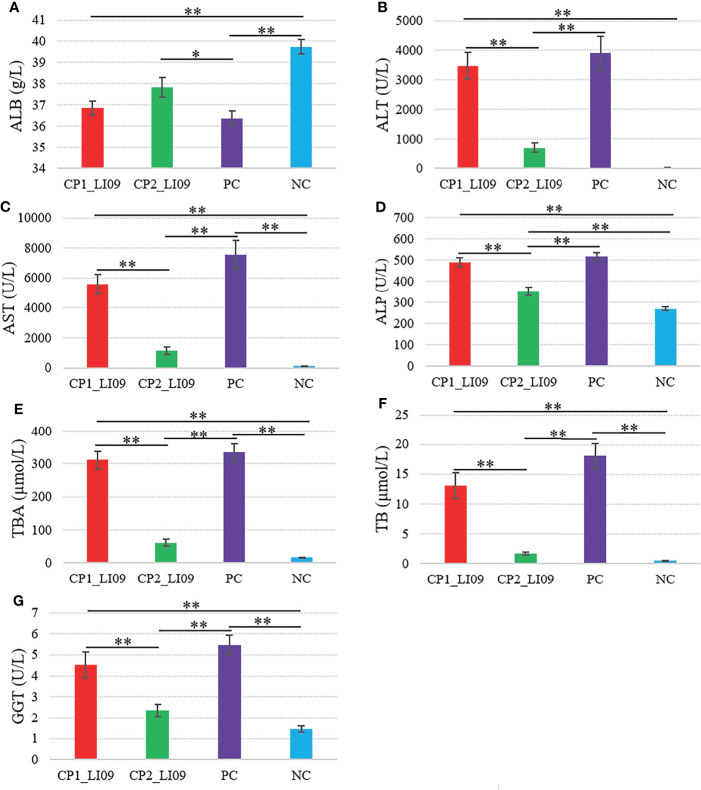
Comparisons of liver function variables in the LI09 cohorts with different cytokine profiles (i.e., CP1_LI09 and CP2_LI09 cohorts) and control cohorts. PC represents positive control; NC represents negative control. **(A)** ALB; **(B)** ALT; **(C)** AST; **(D)** ALP; **(E)** TBA; **(F)** TB; **(G)** GGT. * represented 0.01 < P < 0.05; ** represented P < 0.01.

Six out of the seven measured liver function variables, i.e., ALT, AST, ALP, TBA, TB, and GGT, were greater in the CP1_LI09 cohort than in the CP2_LI09 cohort (**Figure 2**). By contrast, ALB was at similar level between the two cohorts.

**Figure 3:**
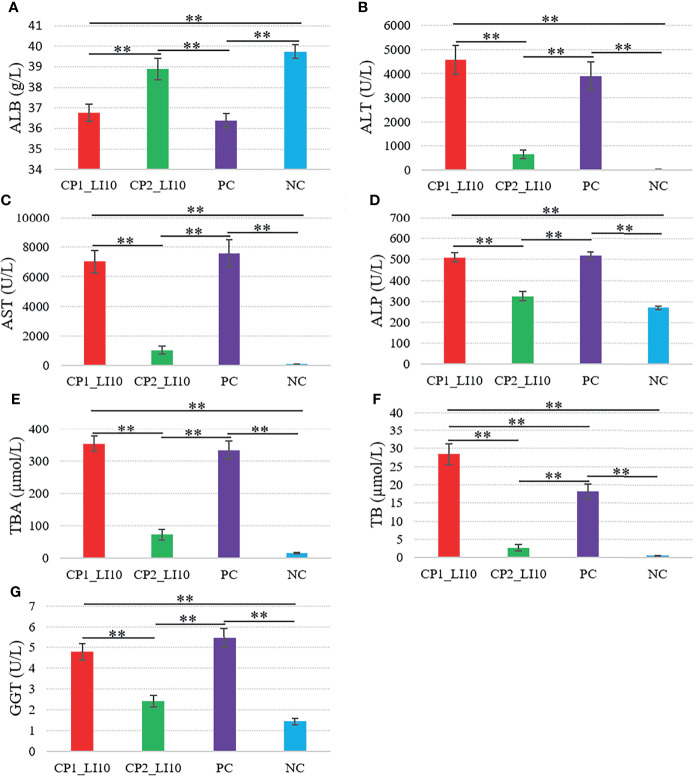
Comparisons of liver function variables in the LI10 cohorts with different cytokine profiles (i.e., CP1_LI10 and CP2_LI10 cohorts) and control cohorts. PC represents positive control; NC represents negative control. **(A)** ALB; **(B)** ALT; **(C)** AST; **(D)** ALP; **(E)** TBA; **(F)** TB; **(G)** GGT. ** represented P < 0.01.

Similarly, ALT, AST, ALP, TBA, TB, and GGT were determined at higher levels in the CP1_LI10 cohort than in the CP2_LI10 cohort (**Figure 3**), while ALB was lower in the CP1_LI10 cohort than in the CP2_LI10 cohort (**Figure 3**).

**Figure 4:**
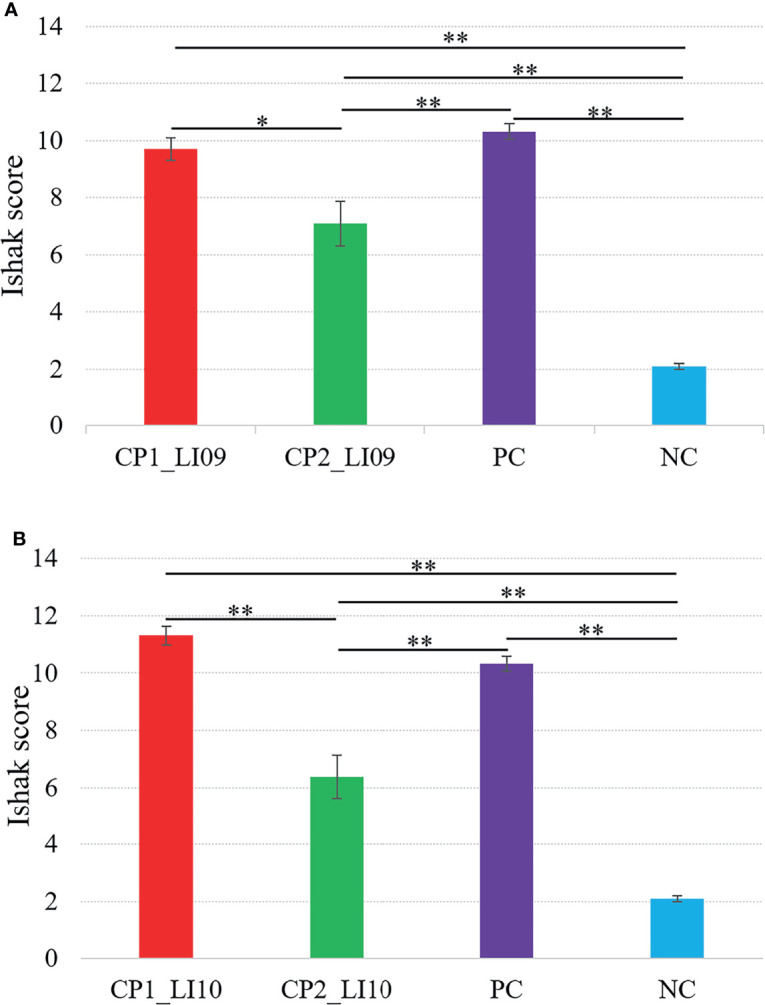
Comparisons of Ishak scores in the **(A)** LI09 cohorts with different cytokine profiles (i.e., CP1_LI09 and CP2_LI09 cohorts) and control cohorts and **(B)** LI10 cohorts with different cytokine profiles (i.e., CP1_LI10 and CP2_LI10 cohorts) and control cohorts. PC represents positive control; NC represents negative control. * represented 0.01 < P < 0.05; ** represented P < 0.01.

Ishak scoring system was used to help evaluate the liver histopathology, and in this study, a higher Ishak score represented greater liver injury severity. The Ishak score was greater in the CP1_LI09 cohort than in the CP2_LI09 cohort (**Figure 4A**), and the same score was greater in the CP1_LI10 cohort than in the CP2_LI10 cohort (**Figure 4B**). The rats with CP1 in LI09 and LI10 cohorts had more severe liver injury than those with CP2, further suggesting that CP2 was the “better cytokine profile” compared with CP1.

### Bacterial Microbiome Composition Analyses

Firmicutes, Bacteroidota, and Verrucomicrobiota were determined as the three most abundant bacterial phyla in the LI09 and LI10 cohorts with different cytokine profiles.

**Figure 5:**
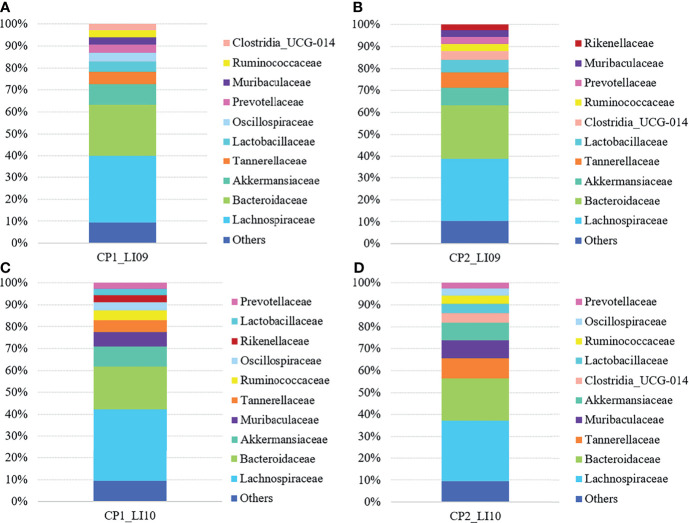
Bacterial family compositions in the LI09 and LI10 cohorts with different cytokine profiles, i.e., **(A)** CP1_LI09, **(B)** CP2_LI09, **(C)** CP1_LI10 and **(D)** CP2_LI10 cohorts.

At the family level, Lachnospiraceae and Bacteroidaceae were determined with the largest abundances in the bacterial microbiome of all the LI09 and LI10 cohorts with different cytokine profiles (**Figure 5**). Akkermansiaceae was the third most abundant bacterial family in CP1_LI09, CP1_LI10, and CP2_LI09 cohorts, while Tannerellaceae was determined with the third most abundance in CP2_LI10 cohort (**Figure 5**).

Shannon and Pielou indices were both similar in bacterial microbiome of the CP1_LI09, CP2_LI09, PC, and NC cohorts (both *p* > 0.09), while a significant difference was determined in observed species of the four cohorts (*p* < 0.001). CP1_LI09 and CP2_LI09 cohorts were found with similar observed species, while they were both greater than in the NC cohort (**Supplementary Figure S2A**). Likewise, Shannon and Pielou indices were both similar between CP1_LI10, CP2_LI10, PC, and NC cohorts (both *p* > 0.05), while observed species was different between the four cohorts (*p* < 0.001). No difference was found in the observed species of CP1_LI10 and CP2_LI10 cohorts, but they were both greater than in the PC and NC cohorts (**Supplementary Figure S2B**).

PERMANOVA analysis suggested that the bacterial composition was similar between CP1_LI09 and CP2_LI09 cohorts (R^2^ = 0.028, *p* > 0.26), and they were both different from the PC and NC cohorts (*p* < 0.002) The same analysis determined a significant difference in the bacterial composition between CP1_LI10 and CP2_LI10 (R^2^ = 0.063, *p*< 0.001), and they were both different from the PC and NC cohorts (*p* < 0.001).

SIMPER analysis determined that the similarity of bacterial phylotype abundances within CP1_LI09 was higher than that of CP2_LI09, i.e., 51% versus 46%. The same analysis determined a dissimilarity of 52% between CP1_LI09 and CP2_LI09 cohorts. Likewise, SIMPER analysis determined that the similarity of bacterial phylotype abundances was greater within CP1_LI10 (i.e., 51%) than within CP2_LI10 (i.e., 44%). The same analysis determined a dissimilarity of 55% between CP1_LI09 and CP2_LI09 cohorts.

**Figure 6:**
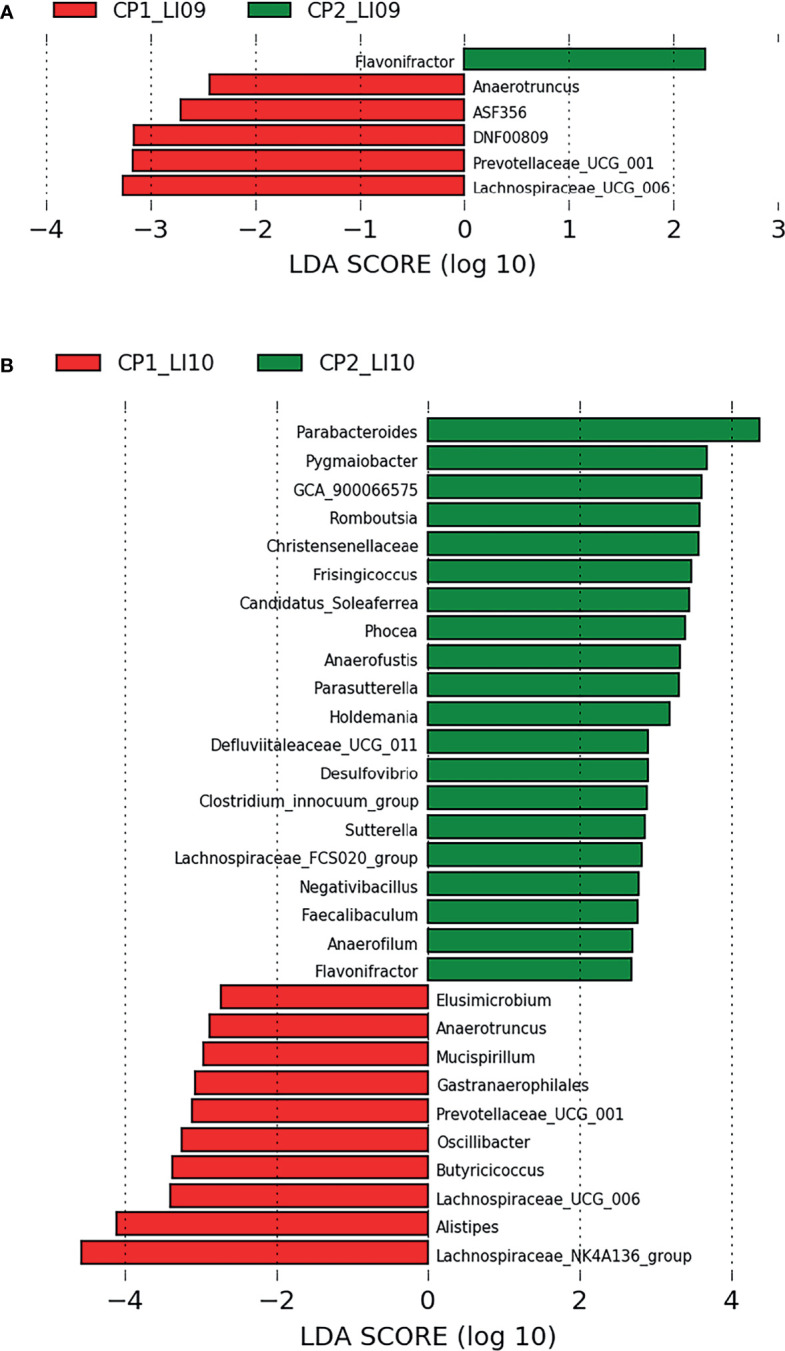
LEfSe analysis determined the bacteria associated with **(A)** LI09 cohorts with different cytokine profiles (i.e., CP1_LI09 and CP2_LI09 cohorts) and **(B)** LI10 cohorts with different cytokine profiles (i.e., CP1_LI10 and CP2_LI10 cohorts).

LEfSe analysis determined five bacteria associated with CP1_LI09 cohort and one bacterium (i.e., *Flavonifractor*) associated with CP2_LI09 cohort (**Figure 6A**), among which *Lachnospiraceae_UCG_006* was most associated with CP1_LI09 cohort. The same analysis revealed that 30 bacteria were associated with CP1_LI10 and CP2_LI10 cohorts (**Figure 6B**), among which *Lachnospiraceae_NK4A136_group* and *Parabacteroides* were most associated with CP1_LI10 and CP2_LI10 cohorts, respectively.

### Fungal Microbiome Composition Analyses

Ascomycota and Basidiomycota were determined as the two most abundant fungal phyla in all the LI09 and LI10 cohorts with different cytokine profiles.

**Figure 7:**
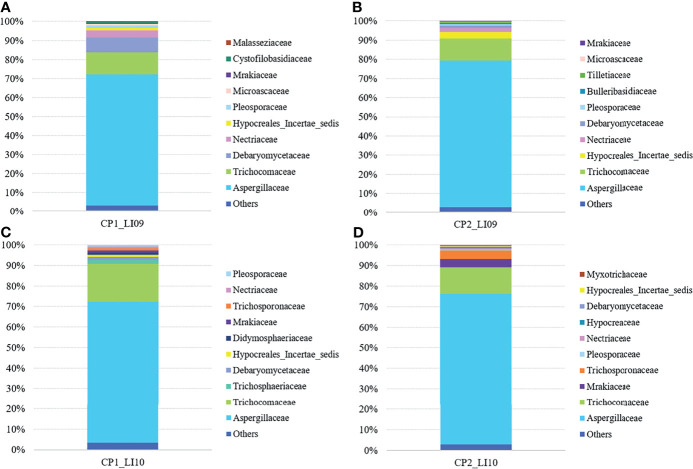
Fungal family compositions in the LI09 and LI10 cohorts with different cytokine profiles, i.e., **(A)** CP1_LI09, **(B)** CP2_LI09, **(C)** CP1_LI10 and **(D)** CP2_LI10 cohorts.

At the family level, Aspergillaceae and Trichocomaceae were determined with most abundances in the LI09 and LI10 cohorts with different cytokine profiles (**Figure 7**). Debaryomycetaceae, Hypocreales_Incertae_sedis, Trichosphaeriaceae, and Mrakiaceae were determined as the third largest abundances in the mycobiome of CP1_LI09, CP1_LI10 and CP2_LI09 and CP2_LI10 cohorts, respectively (**Figure 7**).

Shannon and Pielou indices were both similar in the mycobiome between the CP1_LI09, CP2_LI09, PC, and NC cohorts (both *p* > 0.68), while observed fungal species was different in the four cohorts (*p* < 0.009). No difference was determined in the fungal observed species of CP1_LI09 and CP2_LI09, but they were both less than that in the NC cohort (**Supplementary Figure S3**). Likewise, the three alpha diversity indices were all similar in the mycobiome of CP1_LI10, CP2_LI10, PC, and NC cohorts (all *p* > 0.53).

PERMANOVA analysis revealed that the mycobiome composition was similar between CP1_LI09 and CP2_LI09 cohorts (R^2^ = 0.03, *p* > 0.22), but they were both different from PC and NC cohorts (*p* < 0.004). The same analysis determined a significant difference in the mycobiome composition between CP1_LI10 and CP2_LI10 cohorts (R^2^ = 0.049, *p* < 0.007), and they were both different from PC and NC cohorts (*p* < 0.025).

SIMPER analysis determined that the similarity of fungal phylotype abundances within the CP1_LI09 cohort (i.e., 29%) was lower than that within the CP2_LI09 cohort (i.e., 34%). The same analysis determined a dissimilarity of 69% between CP1_LI09 and CP2_LI09 cohorts. Likewise, SIMPER analysis determined that the similarity of fungal phylotype abundances was lower within the CP1_LI10 cohort than that within the CP2_LI10 cohort, i.e., 34% versus 40%. The same analysis determined a dissimilarity of 65% between CP1_LI09 and CP2_LI09 cohorts.

**Figure 8:**
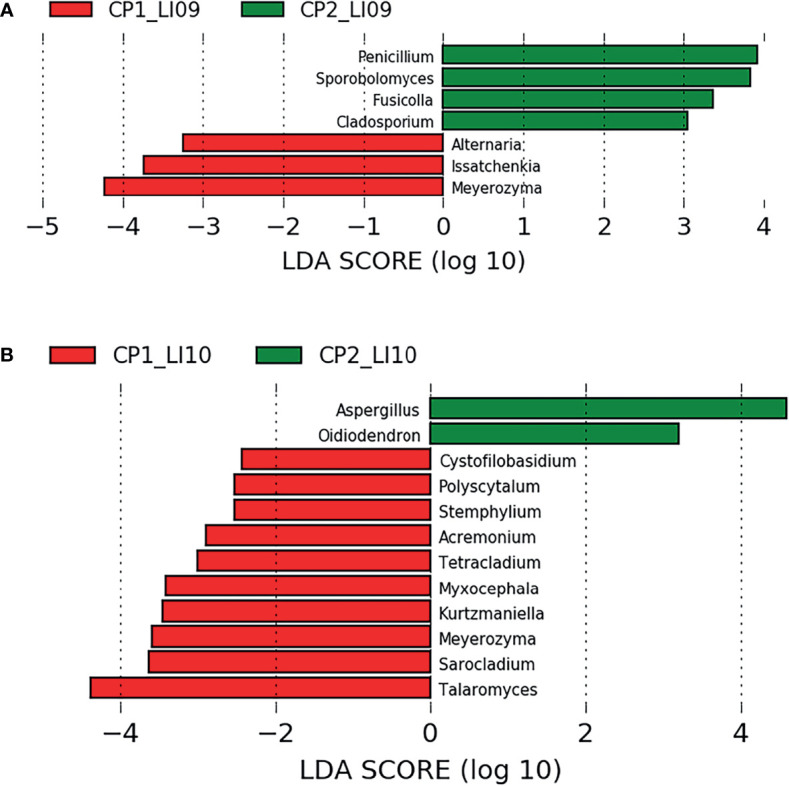
LEfSe analysis determined the fungi associated with **(A)** LI09 cohorts with different cytokine profiles (i.e., CP1_LI09 and CP2_LI09 cohorts) and **(B)** LI10 cohorts with different cytokine profiles (i.e., CP1_LI10 and CP2_LI10 cohorts).

LEfSe analysis determined seven fungi associated with CP1_LI09 and CP2_LI09 cohorts (**Figure 8A**), among which *Meyerozyma* and *Penicillium* were most associated with CP1_LI09 and CP2_LI09 cohorts, respectively. The same analysis determined that 12 fungi were associated with CP1_LI10 and CP2_LI10 cohorts (**Figure 8B**), among which *Talaromyces* and *Aspergillus* were associated with CP1_LI10 and CP2_LI10 cohorts, respectively.

### Effects of Bacteria and Fungi on the Cytokine Profiles

**Figure 9:**
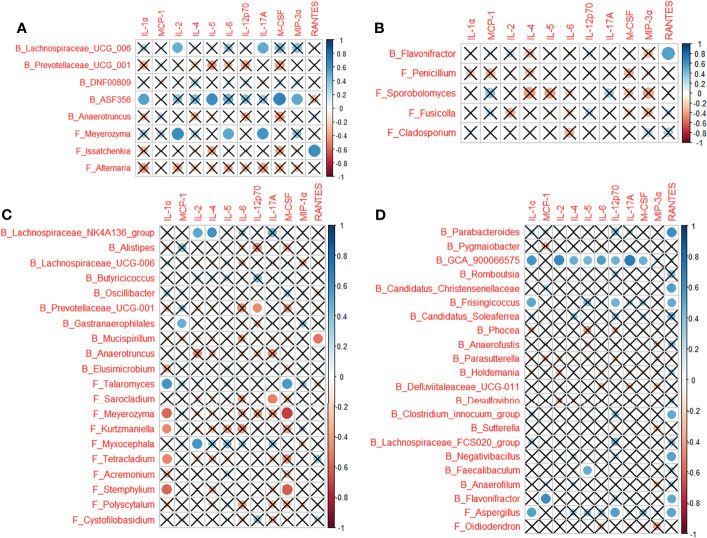
Correlations between cytokines in the cytokine profile and the microbes associated with each of **(A)** CP1_LI09, **(B)** CP2_LI09, **(C)** CP1_LI10, and **(D)** CP2_LI10 cohorts. “B_” and “F_” represent bacteria and fungi, respectively. Cross represents no positive or negative correlation.

Multiple correlations were determined between the cytokines in cytokine profile and the microbes associated with each of the CP1_LI09, CP1_LI10, and CP2_LI10 cohorts, except the CP2_LI09 cohort (**Figure 9**). *ASF356* and *Meyerozyma* were determined with more correlations in the CP1_LI09 cohort (**Figure 9A**). *Lachnospiraceae_NK4A136_group*, *Meyerozyma*, *Stemphylium*, and *Talaromyces* were determined with more correlations with the cytokines in the CP1_LI10 cohort, and *Talaromyces* seemed to have an opposite effect on IL-α and M_CSF when comparing with *Meyerozyma* and *Stemphylium* (**Figure 9C**). *GCA_900066575* was negatively correlated with most cytokines in the cytokine profile of CP2_LI10 cohort (**Figure 9D**).

**Figure 10:**
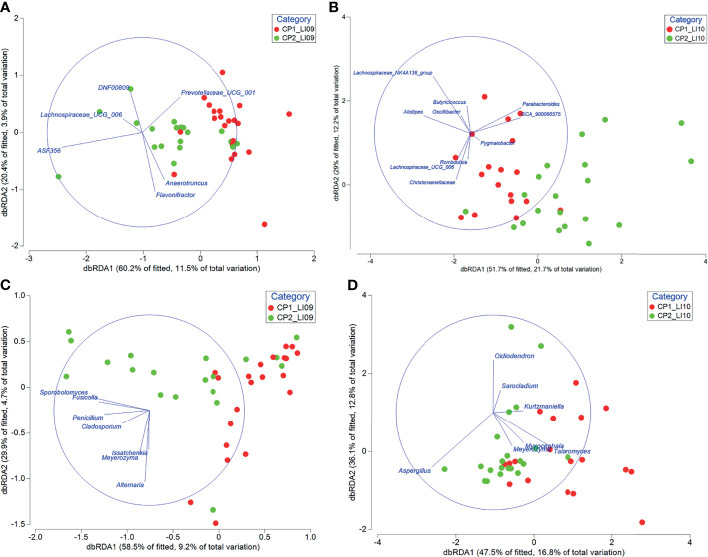
Distance-based redundancy analyses revealed the impact of bacteria associated with **(A)** CP1_LI09 and CP2_LI09 and **(B)** CP1_LI10 and CP2_LI10 cohorts on the cytokine profiles of the corresponding cohorts, and the impact of fungi associated with **(C)** CP1_LI09 and CP2_LI09, and **(D)** CP1_LI10 and CP2_LI10 cohorts on the cytokine profiles of the corresponding cohorts. Up to five bacteria or fungi most associated with each cohort were selected for this analysis.

Some of the bacteria and fungi closely associated with CP1_LI09, CP2_LI09, CP1_LI10, and CP2_LI10 cohorts were determined to influence the cytokine profiles in the corresponding cohorts (**Figure 10**).

### Correlations Between Bacteria and Fungi

Different correlations were determined between the bacteria and fungi associated with CP1_LI09, CP2_LI09, CP1_LI10, or CP2_LI10 cohorts. Three correlations were determined in the bacterial and fungi associated with the CP1_LI09 cohort, i.e., a positive correlation between *Prevotellaceae_UCG_001* and *Meyerozyma*, negative correlations between *Lachnospiraceae_UCG_006* and *Alternaria* and between *ASF356* and *Issatchenkia*. By contrast, no correlation was determined between the bacteria and fungi associated with the CP2_LI09 cohort.

**Figure 11:**
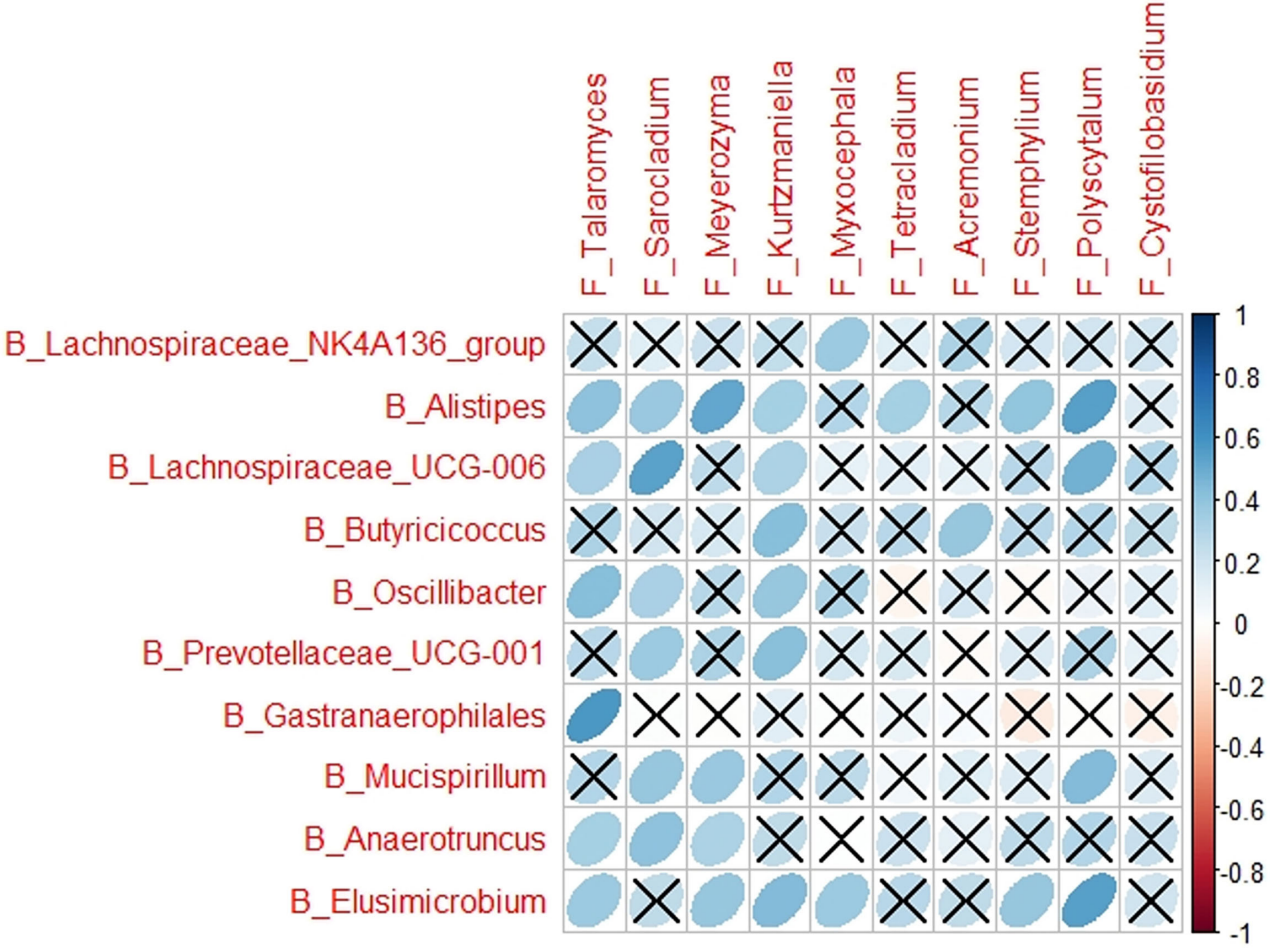
The correlations between bacteria and fungi in the CP1_LI10 cohort.

All the nine correlations were determined to be positive between six bacterial and six fungi associated with the CP1_LI10 cohort (**Figure 11**). *Defluviitaleaceae_UCG_011*, *Candidatus_Christensenellaceae*, and *Pygmaiobacter* were positively correlated with *Oidiodendron* in the CP2_LI10 cohort.

### Microbiological Network Analyses

CoNet results revealed 10 microbes with most correlations in the intestinal microbiome networks of LI09 and LI10 cohorts with different cytokine profiles (**Tables 1**, **2**). Seven out of the top 10 microbes with most correlations in the CP2_LI09 cohort were not determined in the top 10 microbes in the CP1_LI09 cohort (**Table 1**). Likewise, the top eight microbes with most correlations in the CP1_LI10 cohort were all not determined in the top 10 microbes in the CP2_LI10 cohort (**Table 2**). *Eubacterium* and *Clostridium* were both determined with many correlations in the microbiome networks of both CP2_LI09 and CP2_LI10 cohorts but not in the probiotics-treated cohorts with CP1.

**Table 1:** The top 10 microbes with most correlations in the intestinal microbiome networks of the LI09 cohorts with different cytokine profiles, i.e., CP1_LI09 and CP2_LI09 cohorts.

Rank	CP1_LI09 cohort	CP2_LI09 cohort
1	B_*Papillibacter*	B_*Intestinimonas*
2	B_*Corynebacterium*	B_*Papillibacter*
3	B_*Hungatella*	F_Cephalotrichiella*
4	B_*Bacteroides*	B_Eubacterium*
5	F_*Myceliophthora*	B_*Corynebacterium*
6	B_*DNF00809*	B_Bilophila*
7	B_*Intestinimonas*	B_Rikenella*
8	F_*Cystobasidium*	B_Lachnospiraceae_NK4B4_group*
9	F_*Metschnikowia*	B_Aerococcus*
10	F_*Cutaneotrichosporon*	B_Clostridium*

“B_” and “F_” represent the microbes belonging to bacteria and fungi, respectively. Rank represents the rank of correlation number.

*Represents the microbes with most correlations found in CP2_LI09 cohort but not CP1_LI09 cohort.

**Table 2:** The top 10 microbes with most correlations in the intestinal microbiome networks of the LI10 cohorts with different cytokine profiles, i.e., CP1_LI10 and CP2_LI10 cohorts.

Rank	CP1_LI10 cohort	CP2_LI10 cohort
1	B_*Megasphaera*	B_Lachnospiraceae_GCA_900066755*
2	B_*Bifidobacterium*	F_Nothophoma*
3	F_*Blumeria*	B_Eubacterium*
4	B_*Acetobacter*	F_Botrytis*
5	B_*Prevotella*	F_Barnettozyma*
6	F_*Acremonium*	B_Pseudoflavonifractor*
7	F_*Septoria*	B_Azospirillum*
8	F_*Boeremia*	B_Clostridium*
9	F_*Dichotomopilus*	F_*Acremonium*
10	F_*Udeniomyces*	B_*Bifidobacterium*

“B_” and “F_” represented the microbes belonging to bacteria and fungi, respectively. Rank represented the rank of correlation number. *Represented the microbes with most correlations found in CP2_LI10 cohort but not CP1_LI10 cohort.

Among these bacteria and fungi, *Hungatella*, *Papillibacter*, and *Myceliophthora* were determined as gatekeepers in the network of the CP1_LI09 cohort (fragmentation analyses, all *p* < 0.05). In the CP2_LI09 cohort, *Corynebacterium*, *Eubacterium*, *Papillibacter*, and *Cephalotrichiella* were identified as the microbiome network gatekeepers (fragmentation analyses, all *p* < 0.05). By contrast, *Udeniomyces* and *Pseudoflavonifractor* were determined as the only gatekeepers in the microbiome networks of CP1_LI10 and CP2_LI10 cohorts, respectively (fragmentation analyses, both *p* < 0.03).

Fragmentation analysis showed that the fragmentation level of the microbiome network of CP1_LI09 cohort was lower than that of the CP2_LI09 cohort, i.e., 0.497 versus 0.575. Similarly, the fragmentation level was lower in the CP1_LI10 cohort (i.e., 0.416) than that in the CP2_LI10 cohort (i.e., 0.527).

## Discussion

Multiple probiotics have been used to alleviate different types of liver injury (17–19). Liver injury severity, gut microbiome alterations, changes in liver function, and cytokine variables have been used to evaluate the effects of probiotics on the treated cohort (20, 21). Different intestinal microbes could have different abilities of improving these different aspects of the probiotics-treated cohorts. The present study demonstrated the intestinal microbiome associated with distinct cytokine profiles of the LI09 and LI10 pre-treated rats with liver injury and explored the microbes with the biomarker potentials to assist with the evaluation of better immune status in the two probiotics-treated cohorts.

Cytokine profiles have been investigated in the cohorts treated with probiotics (22, 23). In this study, the two cytokine profiles (i.e., CP1 and CP2) were determined by PAM clustering analysis based on the overall pattern of the same 11 cytokines, which were determined with significant different levels among LI09, LI10, PC, and NC cohorts. All rats in PC and NC cohorts were determined with CP1and CP2, respectively, while the rats with CP1 in LI09 and LI10 cohorts were determined with more severe liver injury than those with CP2, suggesting that CP2 represented better immune status and was the “better immune profile” compared with CP1. PAM analysis has been widely used in the microbiome studies to cluster the microbiome in the same or different cohorts (24, 25) but was seldom used to cluster the immune variables.

The cytokines in the cytokine profile in this study, i.e., IL-1α, IL-2, IL-4, IL-5, IL-6, IL-12p70, IL-17A, M-CSF, MCP-1, MIP-3α, and RANTES, were all important to the immune system of mammals (26, 27). Their alterations were associated with the cohorts with different types of liver injury. T-cell activation could be promoted by IL-1α in mice with carbon tetrachloride-induced liver injury (28). IL-2 and IL-17A were independent risk factors that could lead to liver injury in coronavirus disease 2019 (COVID-19) patients at admission (29). Elevated level of plasma IL-4 was determined in the mice with dicloxacillin-induced liver injury (30). Serum IL-5 was increased in the mice with *Schistosoma mansoni*-induced granulomatous liver injury (31). The increased level of serum IL-6 was found in the rats with thioacetamide-induced liver injury (32). DEREG mice with liver fibrosis were found with higher level of IL-12p70, MCP-1, and RANTES (33). M-CSF and MIP-3α were increased in the rats with D-GalN-induced acute liver injury (12).

The majority of the measured liver function variables were determined at higher levels in the LI09 and LI10 cohorts with CP1 (i.e., CP1_LI09 and CP1_LI10 cohorts) than the corresponding cohorts with CP2 (i.e., CP2_LI09 and CP2_LI10 cohorts). As previous study has determined that the majority of liver function variables were lower in the NC cohort than in the PC cohort (12), it suggested that the cohorts with CP2 had better liver function compared with those with CP1. Similarly, Ishak score was greater in CP1_LI09 and CP1_LI10 cohorts than the corresponding cohorts with CP2. As lower Ishak score represented lower liver injury severity (34, 35), it indicated that the cohorts with CP2 profile were with lower liver injury severity.

Bacterial microbiome alterations in probiotics-treated cohorts have been well studied (36, 37). In the current study, PERMANOVA results showed that the compositions of both bacterial and fungal mycobiome were similar between the CP1_L09 and CP2_LI09 cohorts, while the same microbial compositions were different between the CP1_LI10 and CP2_LI10 cohorts. These suggest that the better cytokine profile in the LI10 cohort were associated with the altered bacterial and fungal microbiome compositions. SIMPER analysis has been widely used in the microbiome studies for different objectives (38, 39). SIMPER analyses revealed that the phylotype abundances of bacterial and fungal microbiome were both different between CP1_LI09 and CP2_LI09 cohorts, and between CP1_LI10 and CP2_LI10 cohorts, suggesting that the different cytokine profiles in LI09 and LI10 cohorts were associated with the altered phylotype abundances of the intestinal microbiome.

LEfSe has been carried out in multiple microbiome studies to determine the phylotypes associated with the different cohorts (40, 41). LEfSe analysis determined that multiple bacteria were associated with LI09 cohorts with different cytokine profiles, among which *Lachnospiraceae_UCG_006* and *Flavonifractor* were most associated with CP1_LI09 and CP2_LI09 cohorts, respectively. The enriched *Lachnospiraceae_UCG_006* was associated with the intervention of Nostoc commune Vaucher by polysaccharides (42). *Flavonifractor plautii* was capable of attenuating inflammatory responses in the obese adipose tissue (43). As for fungi, *Meyerozyma* and *Penicillium* were most associated with CP1_LI09 and CP2_LI09 cohorts, respectively. *Meyerozyma* has been found in the patients with vulvovaginal candidiasis infection, while some *Penicillium* species have been used for the beneficial products and cheese-making (44, 45). Some alternative fungi were also closely associated with CP2_LI09 cohort, e.g., *Sporobolomyces* and *Fusicolla*. *Sporobolomyces* could accumulate beneficial metabolites (46), while *Fusicolla* was found as a fungus of soil origin (47).

Likewise, multiple bacteria and fungi were associated with the LI10 cohorts with different cytokine profiles. *Lachnospiraceae_NK4A136_group* and *Talaromyces* were the bacterium and fungus most associated with the CP1_LI10 cohort, while *Parabacteroides* and *Aspergillus* were the bacterium and fungus most associated with the CP2_LI10 cohort. *Lachnospiraceae_NK4A136_group* was determined with low abundance in the obese mice (48), while *Talaromyces* has been found as pathogenic fungus in human beings (49). *Parabacteroides* was a commensal gut bacterium and has been used to alleviate 2,4,6-trinitrobenzene sulfonic acid (TNBS)-induced colitis in mice (50). *Aspergillus* has been found to produce beneficial protease to improve the colonic luminal environment in rats with high-fat diet (51). Some alternative bacteria and fungi were also closely associated with CP2_LI10 cohort, e.g., *Pygmaiobacter*, *GCA_900066575*, and *Oidiodendron*. More abundant *Pygmaiobacter* was found in the intestines of type 2 diabetes mice treated with debranched corn starch (52). Increased gut *GCA_900066575* has been determined in the high-fat diet mice (53), while *Oidiodendron* could improve the root biomass of *Vaccinium corymbosum* (54).

The effect of clinical variables or environmental factors on the microbiome has been well reported (24, 55). However, the potential effect of microbe on the immune profile was seldom studied. In the current study, although the bacteria and fungi associated with each of the four cohort (i.e., CP1_LI09, CP2_LI09, CP1_LI10, and CP2_LI10) were determined to have varied correlations with the individual cytokines, there was no obvious difference in the correlation patterns between the CP1 and CP2 in LI09 and LI10 cohorts. However, the db-RDA results revealed that the microbes associated with each of the four cohorts seemed to influence the corresponding cytokine profiles and were likely to be associated with the formation distinct cytokine profiles. We acknowledge that the detailed mechanisms of the effects of microbes on the cytokine profiles need further investigation.

The correlations between bacteria and fungi were determined in multiple studies for different objectives (56, 57). In this study, different correlations were determined in the LI09 and LI10 cohorts with different cytokine profiles, but it seemed that no obvious difference was found in the correlation patterns or types (i.e., positive and negative) between the different cytokine profiles in the same cohorts.

CoNet and fragmentation analyses have been used to investigate the microbiome networks in multiple studies (16). *Corynebacterium*, *Eubacterium*, *Papillibacter*, and *Cephalotrichiella* were identified as the microbiome network gatekeepers in the CP2_LI09 cohort, while *Pseudoflavonifractor* was the only gatekeeper in the CP2_LI10 cohort. Some *Corynebacterium* and *Eubacterium* species have been determined to have beneficial potentials (58, 59). *Papillibacter* was determined to have the potential to assist *Enterococcus faecium* in enhancing the absorption and utilization of phosphorus (60). *Pseudoflavonifractor* was associated with the regulation of inflammation response in the aged mice with *Listeria monocytogenes* infection (61).

Lower fragmentation levels in the microbiome indicate greater co-occurrence patterns and more biotic interactions (16). The network fragmentation levels of LI09 and LI10 cohorts with CP1 were lower than the corresponding cohorts with CP2, suggesting that more biotic interactions were in the LI09 and LI10 cohorts with CP1 than those with CP2. This could be partly supported by the finding that there are more correlations between bacteria and fungi in the LI09 and LI10 cohorts with CP1 than in the corresponding cohorts with CP2.

In conclusion, the intestinal microbiome associated with distinct cytokine profiles in LI09 and LI10 pre-treated rats with liver injury was characterized. Multiple bacteria and fungi were associated with the better cytokine profiles in LI09 and LI10 cohorts, some of which were determined to influence the cytokine profiles in the corresponding cohorts. Their biomarker potentials in assisting with the evaluation of better cytokine profiles in the probiotics-treated cohorts deserve further investigation.

## Data Availability Statement

The datasets presented in this study can be found in online repositories. The names of the repository and accession number(s) can be found below: https://www.ncbi.nlm.nih.gov/, PRJNA755955 and PRJNA767956.

## Ethics Statement

The animal study was reviewed and approved by Animal Care and Use Committee of the First Affiliated Hospital, Zhejiang University School of Medicine.

## Author Contributions

HZ and LL designed the study. HZ, QL, JX, SL, and RT conducted the experimental work and prepared the dataset. HZ and KC performed data analyses. HZ and LL wrote the manuscript. All research was conducted under supervision of LL. All authors reviewed the manuscript and approved the submission.

## Funding

This work was supported by the National Natural Science Foundation of China (82003441 and 81790631), the Independent Task of State Key Laboratory for Diagnosis and Treatment of Infectious Diseases (2021ZZ15), and the National Key Research and Development Program of China (2018YFC2000500).

## Conflict of Interest

The authors declare that the research was conducted in the absence of any commercial or financial relationships that could be construed as a potential conflict of interest.

## Publisher’s Note

All claims expressed in this article are solely those of the authors and do not necessarily represent those of their affiliated organizations, or those of the publisher, the editors and the reviewers. Any product that may be evaluated in this article, or claim that may be made by its manufacturer, is not guaranteed or endorsed by the publisher.

## References

[1] Horvatits T Drolz A Trauner M Fuhrmann V. Liver Injury and Failure in Critical Illness. Hepatology (2019) 70:2204–15. doi: 10.1002/hep.30824 31215660

[2] Chijioke O Bawohl M Springer E Weber A. Hepatitis E Virus Detection in Liver Tissue From Patients With Suspected Drug-Induced Liver Injury. Front Med (2015) 2:20. doi: 10.3389/fmed.2015.00020 PMC4378310 25870858

[3] Ferrere G Wrzosek L Cailleux F Turpin W Puchois V Spatz M. Fecal Microbiota Manipulation Prevents Dysbiosis and Alcohol-Induced Liver Injury in Mice. J Hepatol (2017) 66:806–15. doi: 10.1016/j.jhep.2016.11.008 27890791

[4] Zhu J Seo J-E Wang S Ashby K Ballard R Yu D. The Development of a Database for Herbal and Dietary Supplement Induced Liver Toxicity. Int J Mol Sci (2018) 19:2955. doi: 10.3390/ijms19102955 PMC6213387 30274144

[5] Vilas-Boas V Gijbels E Jonckheer J De Waele E Vinken M. Cholestatic Liver Injury Induced by Food Additives, Dietary Supplements and Parenteral Nutrition. Environ Int (2020) 136:105422. doi: 10.1016/j.envint.2019.105422 31884416

[6] Dong X Feng X Liu J Xu Y Pan Q Ling Z. Characteristics of Intestinal Microecology During Mesenchymal Stem Cell-Based Therapy for Mouse Acute Liver Injury. Stem Cells Int (2019) 2019:2403793. doi: 10.1155/2019/2403793 30867666 PMC6379839

[7] Xie J Wang W Dong C Huang L Wang H Li C. Protective Effect of Flavonoids From Cyclocarya Paliurus Leaves Against Carbon Tetrachloride-Induced Acute Liver Injury in Mice. Food Chem Toxicol (2018) 119:392–9. doi: 10.1016/j.fct.2018.01.016 29337229

[8] Chen Z-R Jin S-F Ma W-B Jiang R-L. Intestinal Microecology: A Crucial Strategy for Targeted Therapy of Liver Diseases. Hepatobil Pancreat Dis Int (2021) 20. doi: 10.1016/j.hbpd.2021.07.007 34340922

[9] Zhao L Jiang Y Ni Y Zhang T Duan C Huang C. Protective Effects of *Lactobacillus Plantarum* C88 on Chronic Ethanol-Induced Liver Injury in Mice. J Funct Foods (2017) 35:97–104. doi: 10.1016/j.jff.2017.05.017

[10] Neag MA Catinean A Muntean DM Pop MR Bocsan CI Botan EC. Probiotic *Bacillus Spores* Protect Against Acetaminophen Induced Acute Liver Injury in Rats. Nutrients (2020) 12:632. doi: 10.3390/nu12030632 PMC7146158 32120994

[11] Huang L Zhao Z Duan C Wang C Zhao Y Yang G. *Lactobacillus Plantarum* C88 Protects Against Aflatoxin B 1-Induced Liver Injury in Mice *via* Inhibition of NF-κb–Mediated Inflammatory Responses and Excessive Apoptosis. BMC Microbiol (2019) 19:1–9. doi: 10.1186/s12866-019-1525-4 31357935 PMC6664579

[12] Fang D Shi D Lv L Gu S Wu W Chen Y. *Bifidobacterium Pseudocatenulatum* LI09 and *Bifidobacterium Catenulatum* LI10 Attenuate D-Galactosamine-Induced Liver Injury by Modifying the Gut Microbiota. Sci Rep (2017) 7:1–13. doi: 10.1038/s41598-017-09395-8 28821814 PMC5562910

[13] Ishak K. Histological Grading and Staging of Chronic Hepatitis. J Hepatol (1995) 22:696–9. doi: 10.1016/0168-8278(95)80226-6 7560864

[14] Lv L Gu S Jiang H Yan R Chen Y Chen Y. Gut Mycobiota Alterations in Patients With COVID-19 and H1N1 Infections and Their Associations With Clinical Features. Commun Biol (2021) 4:1–11. doi: 10.1038/s42003-021-02036-x 33850296 PMC8044104

[15] Jiang X-W Li Y-T Ye J-Z Lv L-X Yang L-Y Bian X-Y. New Strain of *Pediococcus Pentosaceus* Alleviates Ethanol-Induced Liver Injury by Modulating the Gut Microbiota and Short-Chain Fatty Acid Metabolism. World J Gastroenterol (2020) 26:6224. doi: 10.3748/wjg.v26.i40.6224 33177795 PMC7596634

[16] Wagner Mackenzie B Waite DW Hoggard M Douglas RG Taylor MW Biswas K. Bacterial Community Collapse: A Meta-Analysis of the Sinonasal Microbiota in Chronic Rhinosinusitis. Environ Microbiol (2017) 19:381–92. doi: 10.1111/1462-2920.13632 27902866

[17] Zhang Z Zhou H Bai L Lv Y Yi H Zhang L. Protective Effects of Probiotics on Acute Alcohol-Induced Liver Injury in Mice Through Alcohol Metabolizing Enzymes Activation and Hepatic TNF-α Response Reduction. J Funct Foods (2019) 59:234–41. doi: 10.1016/j.jff.2019.05.018

[18] Azad M Kalam A Sarker M Li T Yin J. Probiotic Species in the Modulation of Gut Microbiota: An Overview. BioMed Res Int (2018) 2018:9478630. doi: 10.1155/2018/9478630 29854813 PMC5964481

[19] Milosevic I Vujovic A Barac A Djelic M Korac M Radovanovic Spurnic A. Gut-Liver Axis, Gut Microbiota, and its Modulation in the Management of Liver Diseases: A Review of the Literature. Int J Mol Sci (2019) 20:395. doi: 10.3390/ijms20020395 PMC6358912 30658519

[20] Yu L Zhao X-K Cheng M-L Yang G-Z Wang B Liu H-J. *Saccharomyces Boulardii* Administration Changes Gut Microbiota and Attenuates D-Galactosamine-Induced Liver Injury. Sci Rep (2017) 7:1–7. doi: 10.1038/s41598-017-01271-9 28465509 PMC5430957

[21] Xue L He J Gao N Lu X Li M Wu X. Probiotics may Delay the Progression of Nonalcoholic Fatty Liver Disease by Restoring the Gut Microbiota Structure and Improving Intestinal Endotoxemia. Sci Rep (2017) 7:1–13. doi: 10.1038/srep45176 28349964 PMC5368635

[22] Azad M Kalam A Sarker M Wan D. Immunomodulatory Effects of Probiotics on Cytokine Profiles. BioMed Res Int (2018) 2018:8063647. doi: 10.1155/2018/8063647 30426014 PMC6218795

[23] Ashraf R Vasiljevic T Day SL Smith SC Donkor O. Lactic Acid Bacteria and Probiotic Organisms Induce Different Cytokine Profile and Regulatory T Cells Mechanisms. J Funct Foods (2014) 6:395–409. doi: 10.1016/j.jff.2013.11.006

[24] Zha H Liu F Ling Z Chang K Yang J Li L. Multiple Bacteria Associated With the More Dysbiotic Genitourinary Microbiomes in Patients With Type 2 Diabetes Mellitus. Sci Rep (2021) 11:1–13. doi: 10.1038/s41598-021-81507-x 33469094 PMC7815922

[25] Chen Y Guo J Shi D Fang D Chen C Li L. Ascitic Bacterial Composition is Associated With Clinical Outcomes in Cirrhotic Patients With Culture-Negative and Non-Neutrocytic Ascites. Front Cell Infect Microbiol (2018) 8:420. doi: 10.3389/fcimb.2018.00420 30555804 PMC6284044

[26] Mulder PP Vlig M Boekema BK Stoop MM Pijpe A Van Zuijlen PP. Persistent Systemic Inflammation in Patients With Severe Burn Injury is Accompanied by Influx of Immature Neutrophils and Shifts in T Cell Subsets and Cytokine Profiles. Front Immunol (2021) 11:621222. doi: 10.3389/fimmu.2020.621222 33584717 PMC7879574

[27] Hwang JH Lee MJ Seok OS Paek YC Cho GJ Seol HJ. Cytokine Expression in Placenta-Derived Mesenchymal Stem Cells in Patients With Pre-Eclampsia and Normal Pregnancies. Cytokine (2010) 49:95–101. doi: 10.1016/j.cyto.2009.08.013 19819721

[28] Lin D Lei L Zhang Y Hu B Bao G Liu Y. Secreted IL-1α Promotes T-Cell Activation and Expansion of CD11b+ Gr1+ Cells in Carbon Tetrachloride-Induced Liver Injury in Mice. Eur J Immunol (2015) 45:2084–98. doi: 10.1002/eji.201445195 25870999

[29] Liao S Zhan K Gan L Bai Y Li J Yuan G. Inflammatory Cytokines, T Lymphocyte Subsets, and Ritonavir Involved in Liver Injury of COVID-19 Patients. Curr Signal Transduct Ther (2020) 5:1–3. doi: 10.1038/s41392-020-00363-9 PMC7599975 33130825

[30] Higuchi S Kobayashi M Yoshikawa Y Tsuneyama K Fukami T Nakajima M. IL-4 Mediates Dicloxacillin-Induced Liver Injury in Mice. Toxicol Lett (2011) 200:139–45. doi: 10.1016/j.toxlet.2010.11.006 21094227

[31] Araújo M Burger E Dias Novaes R Ami Akatuti A Rodrigues MÂ Mendes ACSC. Impact of Paracoccidioides Brasiliensis Coinfection on the Evolution of *Schistosoma Mansoni*-Induced Granulomatous Liver Injury in Mice. BioMed Res Int (2019) 2019:8319465. doi: 10.1155/2019/8319465 31019973 PMC6451801

[32] Abdelaziz RR Elkashef WF Said E. Tranilast Reduces Serum IL-6 and IL-13 and Protects Against Thioacetamide-Induced Acute Liver Injury and Hepatic Encephalopathy. Environ Toxicol Pharmacol (2015) 40:259–67. doi: 10.1016/j.etap.2015.06.019 26164743

[33] Roh YS Park S Lim CW Kim B. Depletion of Foxp3+ Regulatory T Cells Promotes Profibrogenic Milieu of Cholestasis-Induced Liver Injury. Dig Dis Sci (2015) 60:2009–18. doi: 10.1007/s10620-014-3438-2 25416630

[34] Chang X Wang J Chen Y Long Q Song L Li Q. A Novel Nomogram to Predict Evident Histological Liver Injury in Patients With HBeAg-Positive Chronic Hepatitis B Virus Infection. EBioMedicine (2021) 67:103389. doi: 10.1016/j.ebiom.2021.103389 34004423 PMC8141676

[35] Cheng X Wang H Yang J Cheng Y Wang D Yang F. Arctigenin Protects Against Liver Injury From Acute Hepatitis by Suppressing Immune Cells in Mice. BioMed Pharmacother (2018) 102:464–71. doi: 10.1016/j.biopha.2018.03.060 29579707

[36] Wang Y Wu Y Wang B Xu H Mei X Xu X. *Bacillus Amyloliquefaciens* SC06 Protects Mice Against High-Fat Diet-Induced Obesity and Liver Injury *via* Regulating Host Metabolism and Gut Microbiota. Front Microbiol (2019) 10:1161. doi: 10.3389/fmicb.2019.01161 31191487 PMC6547872

[37] Li Y Lv L Ye J Fang D Shi D Wu W. *Bifidobacterium Adolescentis* CGMCC 15058 Alleviates Liver Injury, Enhances the Intestinal Barrier and Modifies the Gut Microbiota in D-Galactosamine-Treated Rats. Appl Microbiol Biotechnol (2019) 103:375–93. doi: 10.1007/s00253-018-9454-y 30345482

[38] Zha H Lu H Wu J Chang K Wang Q Zhang H. Vital Members in the More Dysbiotic Oropharyngeal Microbiotas in H7N9-Infected Patients. Front Med (2020) 7:396. doi: 10.3389/fmed.2020.00396 PMC7433009 32850904

[39] O’Brien CL Kiely CJ Pavli P. The Microbiome of Crohn’s Disease Aphthous Ulcers. Gut Pathog (2018) 10:1–8. doi: 10.1186/s13099-018-0265-6 30337963 PMC6178265

[40] Zha H Chen Y Wu J Chang K Lu Y Zhang H. Characteristics of Three Microbial Colonization States in the Duodenum of the Cirrhotic Patients. Future Microbiol (2020) 15:855–68. doi: 10.2217/fmb-2019-0270 32662659

[41] Wu P Chen Y Zhao J Zhang G Chen J Wang J. Urinary Microbiome and Psychological Factors in Women With Overactive Bladder. Front Cell Infect Microbiol (2017) 7:488. doi: 10.3389/fcimb.2017.00488 29230385 PMC5712163

[42] Guo M Li Z. Polysaccharides Isolated From Nostoc Commune Vaucher Inhibit Colitis-Associated Colon Tumorigenesis in Mice and Modulate Gut Microbiota. Food Funct (2019) 10:6873–81. doi: 10.1039/c9fo00296k 31584586

[43] Mikami A Ogita T Namai F Shigemori S Sato T Shimosato T. Oral Administration of *Flavonifractor Plautii* Attenuates Inflammatory Responses in Obese Adipose Tissue. Mol Biol Rep (2020) 47:6717–25. doi: 10.1007/s11033-020-05727-6 32808115

[44] Ropars J Didiot E de la Vega RCR Bennetot B Coton M Poirier E. Domestication of the Emblematic White Cheese-Making Fungus *Penicillium Camemberti* and its Diversification Into Two Varieties. Curr Biol (2020) 30:4441–53. doi: 10.1016/j.cub.2020.08.082 32976806

[45] Srinivasan R Prabhu G Prasad M Mishra M Chaudhary M Srivastava R. Penicillium. In: Beneficial Microbes in Agro-Ecology. London: Elsevier (2020). p. 651–67.

[46] Tong X Wang K Chen Z Wang L Xiang T. Endangerment of *Ostrya Rehderiana* Chun and its Relationship With Rhizosphere Soil Microflora. Agron J (2021) 113:746–59. doi: 10.1002/agj2.20451

[47] Wang M Xue J Ma J Feng X Ying H Xu H. *Streptomyces Lydicus* M01 Regulates Soil Microbial Community and Alleviates Foliar Disease Caused by *Alternaria Alternata* on Cucumbers. Front Microbiol (2020) 11:942. doi: 10.3389/fmicb.2020.00942 32499771 PMC7243425

[48] Ma L Ni Y Wang Z Tu W Ni L Zhuge F. Spermidine Improves Gut Barrier Integrity and Gut Microbiota Function in Diet-Induced Obese Mice. Gut Microbes (2020) 12:1832857. doi: 10.1080/19490976.2020.1832857 PMC7668533 33151120

[49] Chan JF Lau SK Yuen K-Y Woo PC. *Talaromyces* (*Penicillium*) Marneffei Infection in Non-HIV-Infected Patients. Emerg Microbes Infect (2016) 5:1–9. doi: 10.1038/emi.2016.18 PMC4820671 26956447

[50] Cuffaro B Assohoun AL Boutillier D Súkeníková L Desramaut J Boudebbouze S. *In Vitro* Characterization of Gut Microbiota-Derived Commensal Strains: Selection of *Parabacteroides Distasonis* Strains Alleviating TNBS-Induced Colitis in Mice. Cells (2020) 9:2104. doi: 10.3390/cells9092104 PMC7565435 32947881

[51] Yang Y Sitanggang NV Kato N Inoue J Murakami T Watanabe T. Beneficial Effects of Protease Preparations Derived From *Aspergillus* on the Colonic Luminal Environment in Rats Consuming a High-Fat Diet. BioMed Rep (2015) 3:715–20. doi: 10.3892/br.2015.490 PMC4535064 26405551

[52] Wang Y Ning Y Yuan C Cui B Liu G Zhang Z. The Protective Mechanism of a Debranched Corn Starch/Konjac Glucomannan Composite Against Dyslipidemia and Gut Microbiota in High-Fat-Diet Induced Type 2 Diabetes. Food Funct (2021) 12:9273–85. doi: 10.1039/d1fo01233a 34606538

[53] Li H Liu F Lu J Shi J Guan J Yan F. Probiotic Mixture of *Lactobacillus Plantarum* Strains Improves Lipid Metabolism and Gut Microbiota Structure in High Fat Diet-Fed Mice. Front Microbiol (2020) 11:512. doi: 10.3389/fmicb.2020.00512 32273874 PMC7113563

[54] Bizabani C Fontenla S Dames JF. Ericoid Fungal Inoculation of Blueberry Under Commercial Production in South Africa. Sci Hortic (2016) 209:173–7. doi: 10.1016/j.scienta.2016.06.029

[55] Hermans SM Buckley HL Case BS Curran-Cournane F Taylor M Lear G. Using Soil Bacterial Communities to Predict Physico-Chemical Variables and Soil Quality. Microbiome (2020) 8:1–13. doi: 10.1186/s40168-020-00858-1 32487269 PMC7268603

[56] Mackenzie BW Chang K Zoing M Jain R Hoggard M Biswas K. Longitudinal Study of the Bacterial and Fungal Microbiota in the Human Sinuses Reveals Seasonal and Annual Changes in Diversity. Sci Rep (2019) 9:1–10. doi: 10.1038/s41598-019-53975-9 31758066 PMC6874676

[57] Lemoinne S Kemgang A Belkacem KB Straube M Jegou S Corpechot C. Fungi Participate in the Dysbiosis of Gut Microbiota in Patients With Primary Sclerosing Cholangitis. Gut (2020) 69:92–102. doi: 10.1136/gutjnl-2018-317791 31003979

[58] Colombo M Castilho N Todorov S Nero L. Beneficial and Safety Properties of a *Corynebacterium Vitaeruminis* Strain Isolated From the Cow Rumen. Probiot Antimicrob (2017) 9:157–62. doi: 10.1007/s12602-017-9263-0 28258546

[59] Jones RB Alderete TL Kim JS Millstein J Gilliland FD Goran MI. High Intake of Dietary Fructose in Overweight/Obese Teenagers Associated With Depletion of *Eubacterium* and *Streptococcus* in Gut Microbiome. Gut Microbes (2019) 10:712–9. doi: 10.1080/19490976.2019.1592420 PMC6866686 30991877

[60] Wang W Cai H Zhang A Chen Z Chang W Liu G. *Enterococcus Faecium* Modulates the Gut Microbiota of Broilers and Enhances Phosphorus Absorption and Utilization. Animals (2020) 10:1232. doi: 10.3390/ani10071232 PMC7401662 32698425

[61] Alam MS Gangiredla J Hasan NA Barnaba T Tartera C. Aging-Induced Dysbiosis of Gut Microbiota as a Risk Factor for Increased Listeria Monocytogenes Infection. Front Immunol (2021) 12:672353. doi: 10.3389/fimmu.2021.672353 33995413 PMC8115019

