# Supplementary material for: Characterising the Intestinal Bacterial and Fungal Microbiome Associated With Different Cytokine Profiles in Two Bifidobacterium strains Pre-Treated Rats With D-Galactosamine-Induced Liver Injury
Source: Front Immunol. 2022 Mar 24;13:791152. doi: 10.3389/fimmu.2022.791152 (PMC8987000; doi:10.3389/fimmu.2022.791152)

Supplemental Fig. S1. The cytokines with significant differences in LI09, LI10, PC and NC cohorts, i.e., (A) IL-1α, (B) MCP-1, (C) IL-2, (D) IL-4, (E) IL-5, (F) IL-6, (G) IL-12p70, (H) IL-17A, (I) M-CSF, (J) MIP-3α, (K) RANTES. Note: Further comparisons were conducted between probiotic cohorts (LI09 and LI10) and PC cohorts. ** represented *P* < 0.01; * represented 0.01 < *P* < 0.05; the unit for all the values was pg/ml.


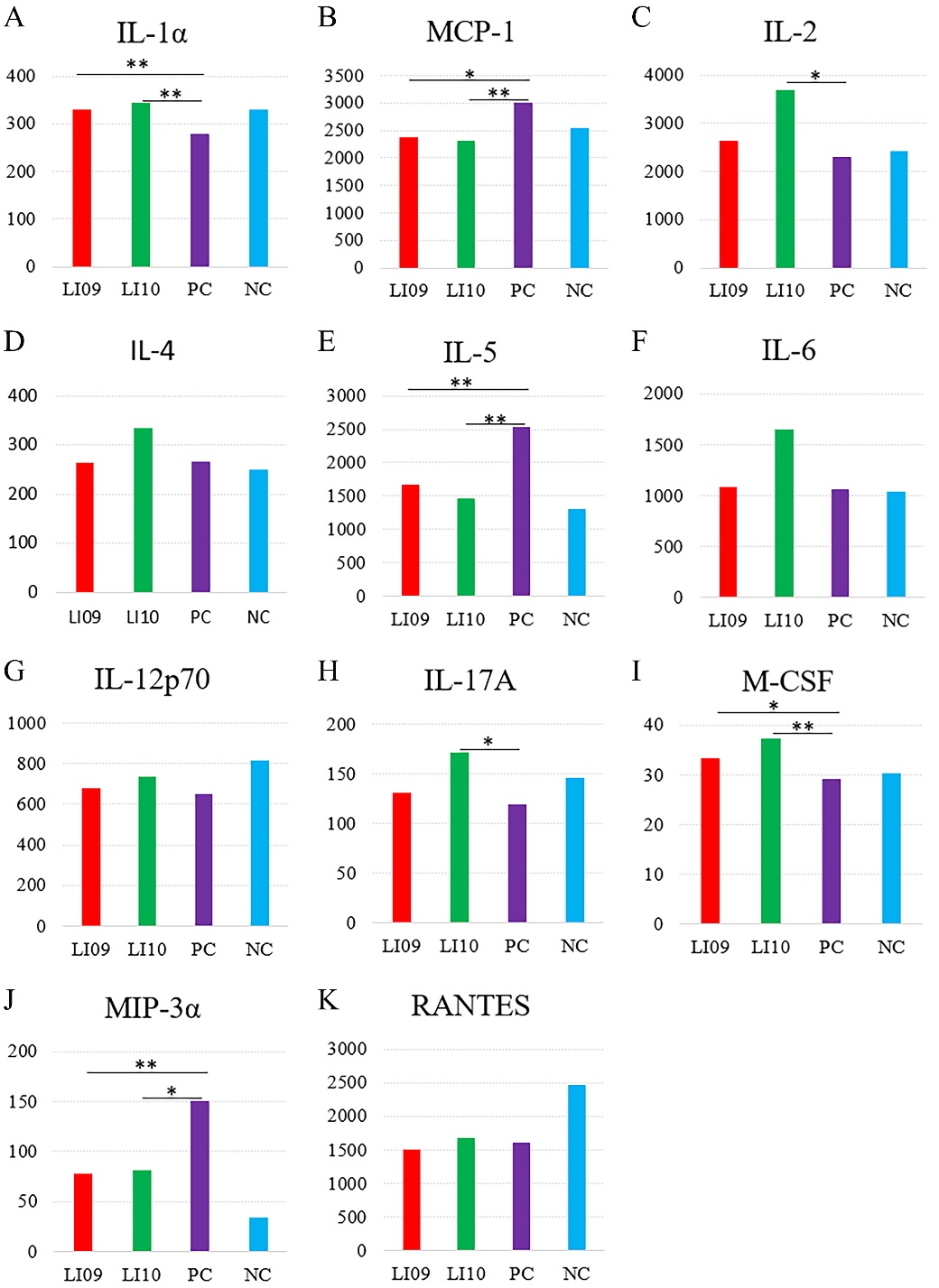


Supplemental Fig. S2. Comparisons of richness index in the bacterial microbiome of (A) LI09 cohort and (B) LI10 cohorts with different cytokine profiles, and their control cohorts. Note: PC represented positive control; NC represented negative control. Note: ** represented *P* < 0.01; * represented 0.01 < *P* < 0.05.


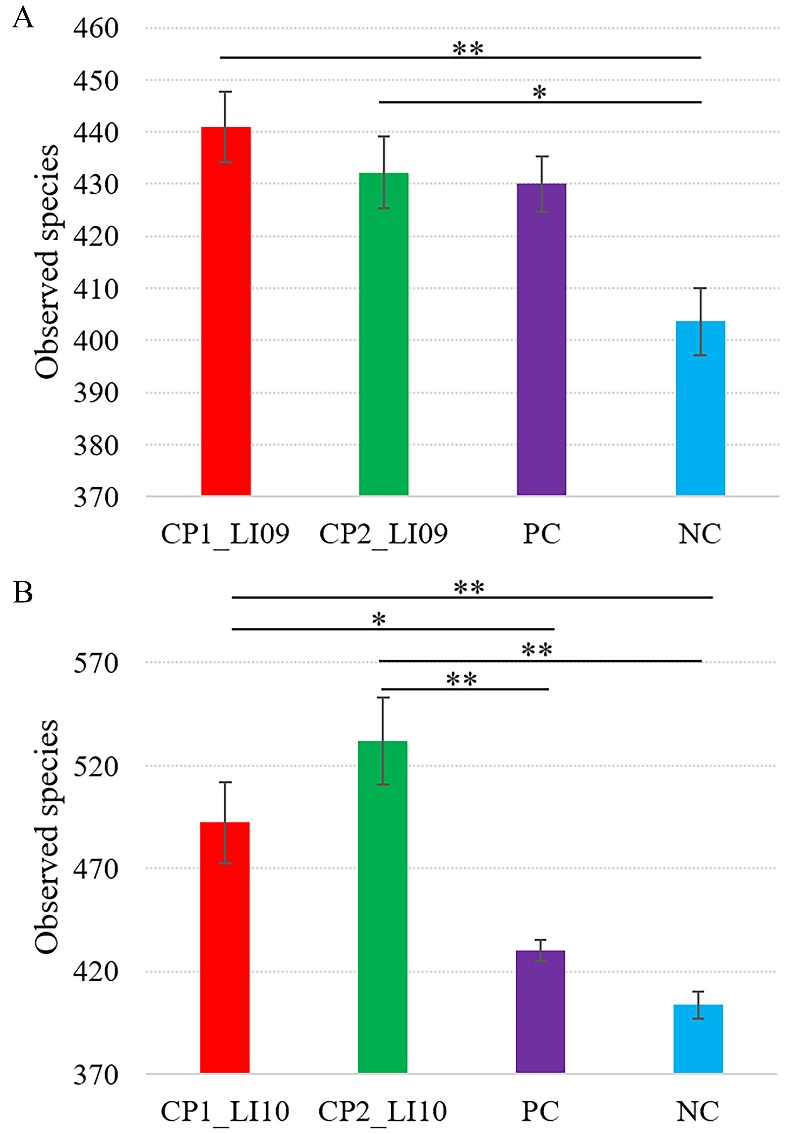


Supplemental Fig. S3. Comparisons of richness index in the fungal microbiome of LI09 cohort with different cytokine profiles, and their control cohorts. Note: PC represented positive control; NC represented negative control. Note: * represented 0.01 < *P* < 0.05.


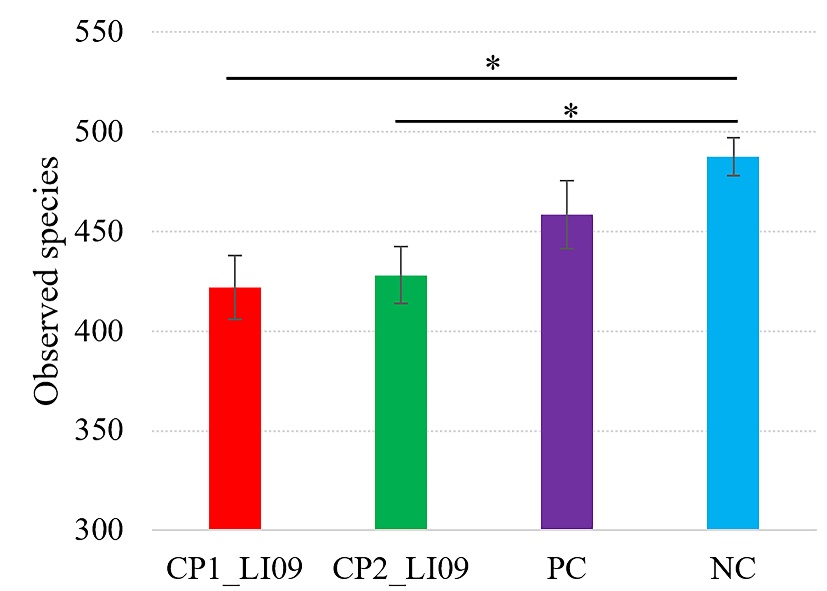

Supplement: Supplementary file 1 [file DataSheet_1.docx]
